# Electrochemical Discrimination of Salbutamol from Its Excipients in Ventolin^TM^ at Nanoporous Gold Microdisc Arrays

**DOI:** 10.3390/s21123975

**Published:** 2021-06-09

**Authors:** Lorraine C. Nagle, Amelie Wahl, Vladimir Ogourstov, Ian Seymour, Fiona Barry, James F. Rohan, Ronan Mac Loughlin

**Affiliations:** 1 Tyndall National Institute, Lee Maltings Complex, University College Cork, T12 R5CP Cork, Ireland; amelie.wahl87@gmail.com (A.W.); vladimir.ogourstov@tyndall.ie (V.O.); ian.seymour@tyndall.ie (I.S.); fiona.barry@tyndall.ie (F.B.); james.rohan@tyndall.ie (J.F.R.); 2 Research and Development, Science and Emerging Technologies, Aerogen Ltd. Galway Business Park, Dangan, H91 EH6C Galway, Ireland; RMacLoughlin@aerogen.com

**Keywords:** nanoporous gold, electrochemical sensor, inhalable pharmaceutical, salbutamol, nanoconfinement effect, selective discriminative amplification, excipient

## Abstract

The emergence of specific drug–device combination products in the inhalable pharmaceutical industry demands more sophistication of device functionality in the form of an embedded sensing platform to increase patient safety and extend patent coverage. Controlling the nebuliser function at a miniaturised, integrated electrochemical sensing platform with rapid response time and supporting novel algorithms could deliver such a technology offering. Development of a nanoporous gold (NPG) electrochemical sensor capable of creating a unique fingerprint signal generated by inhalable pharmaceuticals provided the impetus for our study of the electrooxidation of salbutamol, which is the active bronchodilatory ingredient in Ventolin^TM^ formulations. It was demonstrated that, at NPG-modified microdisc electrode arrays, salbutamol is distinguishable from the chloride excipient present at 0.0154 M using linear sweep voltammetry and can be detected amperometrically. In contrast, bare gold microdisc electrode arrays cannot afford such discrimination, as the potential for salbutamol oxidation and chloride adsorption reactions overlap. The discriminative power of NPG originates from the nanoconfinement effect for chloride in the internal pores of NPG, which selectively enhances the electron transfer kinetics of this more sluggish reaction relative to that of the faster, diffusion-controlled salbutamol oxidation. Sensing was performed at a fully integrated three-electrode cell-on-chip using Pt as a quasi-reference electrode.

## 1. Introduction

Salbutamol, 2-(*tert*-butylamino)-1-[4-hydroxy-3-(hydroxymethyl)phenyl] ethanol (also known as Albuterol), is a *β*2-adrenergic bronchodilator widely used in treating respiratory conditions including bronchial asthma, emphysema, allergen asthma and chronic obstructive pulmonary disease (COPD) [1,2,3]. It is amongst the top ten prescribed drugs in the United States [4] and is usually administered by pressurized metered dose inhaler or nebulizer. It is also an important tool in the development and validation of respiratory drug delivery systems [5,6,7,8] and surrogate tracer aerosol for expensive and hard-to-quantify pharmaceuticals. In vivo, the inhaled dose is absorbed in the lung, with the free drug and its metabolite being eliminated by renal excretion [9]. Unsurprisingly, residues of salbutamol and its metabolites have been found in natural bodies of water, which poses a threat to human health and the environment [10]. Most recently, Palacios-Arreola et al. reported an electrochemical degradation method seeking to address problems incurred by the persistence of salbutamol residues in water bodies [11]. Although salbutamol was originally developed for the treatment of respiratory illness, it has also been used to improve lean meat yield in animals by enhancing growth rate and reducing carcass fat [12]. Consequently, it may accumulate in animal tissues and persist in meat products, with human ingestion of such contaminated foods potentially resulting in acute toxic responses, such as cardiac palpitation, muscle tremors, tachycardia and elevated blood sugar levels [13,14]. All β-agonists, including salbutamol, are banned in the European Union as feed additives for growth promotion in animals, but the illegal use of salbutamol still occurs in many areas across the world. There is a clear need to establish rapid, portable and accurate methods to monitor salbutamol, primarily for its treatment of lung disease but also due to its illicit use in animal husbandry and subsequent accumulation in water bodies.

Salbutamol is usually manufactured and distributed as salbutamol sulphate, which constitutes the active pharmaceutical ingredient (API) in a number of commercially available, quick-relief drug formulations and is marketed as Ventolin^TM^, amongst other brand names. Salbutamol-based drugs are available in inhalable, oral and injectable formulations. Administration using inhalers, or, more frequently, using nebulisers, is most widely adopted, as it is more effective, acts faster, causes fewer side effects and requires lower doses while lasting about as long as the other forms. More recently, the release of fugitive medical aerosol emissions to the local environment and the consequent risk to bystanders in the home and in clinical settings has come into focus [15,16,17,18,19]. With the reported low acceptable occupational exposure limits [20,21] (inhalation and dermal exposure), detection of fugitive pharmaceutical agents may have applications.

Upon administration for respiratory conditions, salbutamol rapidly activates β2-adrenoreceptors, stimulating relaxation of bronchial tubes and dilation of the airways. However, there is a risk to the patient for overuse of salbutamol-based medication, which may induce harmful effects such as reducing its future efficacy. Moreover, salbutamol can induce side effects, particularly at high doses, including headache, anxiety, dry mouth, tachycardia and cardiac arrhythmia [22]. Treatment depends on a patient’s age, their type of respiratory condition and efficacy. It is important to monitor both dosage levels and regimen to ensure patients’ safety. In this regard, drug regulators worldwide indicate that specific drug–device combination nebulisers are preferred over the more common open-label nebulisers for increased control over dosing reproducibility and reliability in order to enhance patients’ safety [23,24]. Desirable features include a lock-out system control to prevent overdosing and signals to confirm dose delivery to the patient. Globally, the drug–device combination products market was valued in 2017 at $81,374 million and is projected to reach $139,193 million by 2025 [25]. However, current technology fails to provide this crucial sensing/monitoring, and achieving sensor compatibility with existing nebuliser settings may be challenging.

Several analytical methods have been successfully applied to the determination of salbutamol, in particular high performance liquid chromatography [26,27] and liquid chromatography [28,29] but also spectrophotometry [30,31], thin layer chromatography [32] and immunoassay [10,33]. Some methods did not achieve sufficient limit of detection or selectivity, yet liquid chromatography can determine trace levels quantitatively with high precision. However, these methods mostly require expensive and bulky instrumentation that is confined to lab-based use, feature complex sample pretreatment procedures, involve long analysis time and are practically impossible to integrate within the controller in a specific drug–device combination that regulates nebuliser function.

Electrochemical sensors operate on the premise that, when an analyte comes in contact with an electrode surface, it undergoes a redox reaction, which, in turn, produces an electrical signal that can then be attributed to the specific analyte. The response of salbutamol on conventional working electrodes is weak, and the sensitivity is low for direct analysis in biological matrices. To this end, nanomaterial-based sensors have attracted considerable interest due to their enlarged electrode surface area and ability to enhance electron transfer. Detection of salbutamol at several electrochemical sensors [1,34,35,36,37,38,39,40,41,42,43] has been reported and is summarized in Table 1. A significant drawback of these is that most are based on modified electrodes, which use expensive modifiers and involve lengthy modification steps. The nanoparticle instability in the case of several modified electrodes leads to their coalescence, making it difficult to achieve a stable and reproducible electrochemical surface area. Moreover, the contribution of the chloride excipient in Ventolin^TM^ formulations to the salbutamol signal was not, to the best of our knowledge, evaluated at the electrochemical sensors developed to date.

Interestingly, although electrochemical sensors offer high sensitivity, portable screening with low sample/reagent consumption, rapid response time and, crucially, have the potential to be embedded in a nebuliser, they have not yet been exploited in increasing the functionality of inhalable drug–device combination products. However, given that the use of wearable electrochemical sensors for monitoring therapeutic and illicit drugs is well established, it is plausible that their integration could ensue in such products [46,47,48]. The development of an electrochemical-based controller capable of accurately detecting whether or not the appropriate medication has been placed into a nebuliser’s medication reservoir must meet certain criteria. Some of the key challenges include (i) the ability of an electrode surface to distinguish API(s) in a pharmaceutical formulation from its excipients, (ii) developing a fabrication procedure that reliably and repeatedly produces the desired electrochemically active surface and (iii) devising novel algorithms to define functionality.

Herein, we demonstrate the application of NPG to salbutamol determination in Ventolin^TM^ formulations with successful discrimination of the two signals associated with salbutamol oxidation and chloride adsorption. NPG is a promising electrochemical sensing platform candidate primarily due to its high specific surface area, originating from its large internal surface area, coupled to its superior catalytic properties [49,50,51,52,53,54] and excellent conductivity [55]. NPG is characterised by a sponge-like 3D network composed of interconnecting nanopores and nanoligaments and was shown to be as strong as bulk gold [56]. Its bicontinuous structure with a unique, highly curved morphology has a high density of steps, holes and kinks, which are populated by low coordination atoms (in contrast to higher coordination atoms present in a close-packed surface of bulk metal). Lower coordination gold atoms interact more strongly with target molecules due to a narrower d-band and local upshift, which are typically involved in Au bonding [57,58] and constitute the active sites in NPG. Recently, Welch et al. [59] identified the stepped sites and grain boundaries inherent in NPG’s highly curved ligaments as the active sites in the electrochemical reduction of CO_2_. Furthermore, NPG is also chemically inert, corrosion resistant and biocompatible [60,61].

NPG is easier to prepare and is more stable than nanoparticle-based catalysts, which are prone to undergo aggregation, causing subsequent loss of available surface area and catalytic activity. Moreover, the effective utilization of nanoparticles requires the additional step of their immobilization on an electrode surface, which in turn may be hampered by delamination and surface area loss. Based on its aforementioned attractive characteristics, NPG is an ideal candidate as an electrochemical sensor [62,63]. It has been successfully exploited in sensing several important gas-phase (e.g., oxygen, carbon monoxide, carbon dioxide, hydrogen peroxide, nitric oxide, hydrazine) and liquid-phase analytes (e.g., glucose and other saccharides, ascorbic acid, dopamine, nitrite, sulphite, heavy metals, BPA, ammonia borane and borohydride), which are of particular relevance in medical [64,65,66,67], environmental [68,69], food safety [70,71] and energy applications, including fuel cells [54,72,73,74,75,76,77,78].

Herein, we report on the fabrication of fully integrated three-electrode cell-on-chip with arrays of recessed gold microdiscs as the working electrode and platinum as the on-chip counter and pseudo-reference electrodes. The reproducible fabrication of NPG overlayers by electrodeposition of a silver–gold alloy in the array electrodes, followed by selective etching of the less noble silver component in nitric acid to create NPG is demonstrated. Finally, we show that such NPG-modified microdisc array electrodes can be applied to selectively detect and quantify salbutamol in Ventolin^TM^ formulations without interference from the chloride excipient, which is not achievable at unmodified bare gold microdisc array electrodes. The single peak in the linear sweep voltammogram observed at bare gold microdisc array electrodes emanates from the overlap in potential for salbutamol oxidation and chloride adsorption reactions; however, the unique nanogeometrical architecture of NPG offers increased selectivity in differentiation of two peaks through (i) exploitation of the nanoconfinement effect at NPG and (ii) the differing electron transfer kinetics of the two reactions. To the best our knowledge, this is the first example of an electrochemical sensor that successfully detected and discriminated salbutamol and chloride signals in Ventolin^TM^ formulations.

## 2. Materials and Methods

### 2.1. Fabrication of Recessed Gold Microdisc Electrode Arrays

Recessed gold microdisc array working electrodes, platinum counter and pseudo-reference electrodes were fabricated using standard photolithographic techniques on four-inch silicon wafer substrates with a 1 µm layer of thermally grown silicon dioxide insulator (Si/SiO_2_) to avoid short circuiting in the metal layers. In this approach, Si/SiO_2_ wafers were spin-coated with a bilayer photoresist process (LORA3A and S1805) to facilitate metal lift-off and exposed to UV light for 4 s through a mask containing the pattern for the counter and pseudo-reference electrodes. A 20 nm thick titanium adhesion layer, followed by a 100 nm thick platinum layer, was then deposited by evaporation, followed by a wet lift-off process (immersion in 1165 resist remover at 90 °C) to remove the photoresist from unpatterned areas so as to obtain the desired platinum structures (about 3.3 cm^2^ each). Alignment marks were patterned along with this first metal layer in order to allow accurate positioning of subsequent masks. Gold microdisc array working electrodes, interconnection tracks and peripheral electrical contact pads (about 1.5 mm in diameter) were then fabricated in a second photolithographic process, whereby a subsequent photoresist layer was patterned by photolithography using a second mask, followed by metal evaporation (titanium/gold 20/100 nm) and the same wet lift-off procedure. Finally, to prevent unwanted electrochemical reactions occurring between metal interconnection tracks and electrochemically active species, ca. 730 nm thick silicon nitride passivation layer was deposited by low pressure chemical vapour deposition onto the wafer surface.

**Figure 1:**
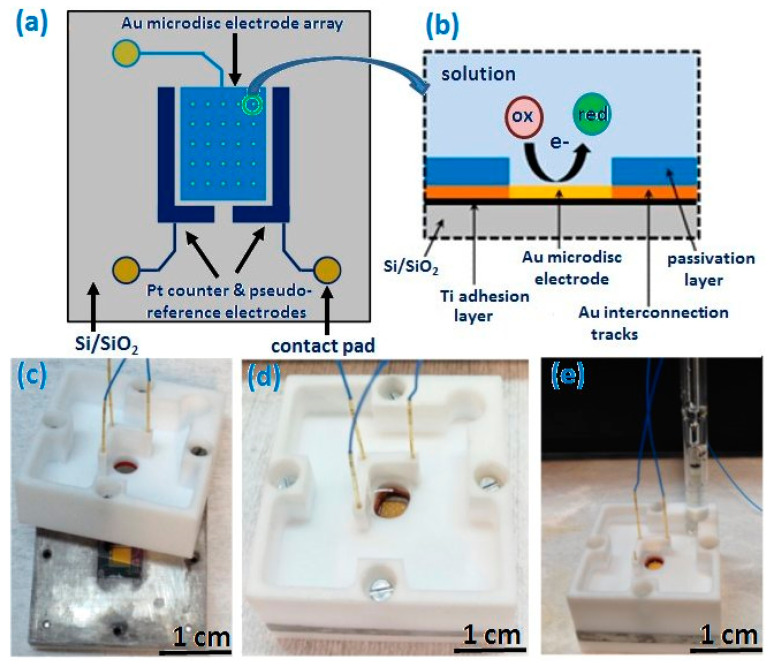
(**a**) Schematic representation of the chip design comprising gold microdisc array working electrodes, platinum counter and pseudo-reference electrodes; micron scale gold interconnection tracks and contact pads; (**b**) cross-sectional representation of a gold microdisc electrode on a chip; (**c**) photograph of a fully fabricated chip mounted in the aluminium base of the custom-designed cell (the cell’s upper PTFE housing includes spring-loaded probes enabling electrical contact between on-chip electrodes and a potentiostat); (**d**,**e**) photographs of a fully assembled chip using on-chip platinum pseudo-reference electrode and an external reference electrode, respectively.

A third photolithographic process, consisting of another photoresist layer patterned by photolithography using a third mask, followed by dry etching (in a CF_4_ plasma), was then carried out to selectively open windows so as to only expose gold microdisc array electrodes, platinum counter and pseudo-reference electrodes to allow exclusive contact between them and a solution of interest. Openings were also patterned above peripheral contact pads to permit electrical contact. Finally, the photoresist was stripped in 1165 resist remover. Following fabrication, wafers were diced into 10.1 × 10.1 mm^2^ chips. Each chip contained an array of 56 contacted gold microdisc working electrodes (23 µm in diameter and separated by 500 µm). Each chip also included a platinum counter electrode and platinum pseudo-reference electrode, located on either side of the array. Figure 1a is a schematic representation overview of the chip design, and Figure 1b shows a cross-sectional representation of a fully integrated gold microdisc electrode in contact with a solution.

### 2.2. Structural and Elemental Characterisation

The dimensions and morphologies of gold microdisc array electrodes and NPG-modified arrays were characterised using a calibrated scanning electron microscope (FEI Quanta FEG 650 SEM) at accelerating voltage of 30 kV. The topography and the surface roughness of gold microdiscs were investigated using a calibrated atomic force microscope (AFM; Dimension 3100, Veeco Instruments Inc., Oyster Bay, NY, USA) in tapping-mode with commercial tapping mode probes (MP-11100, Veeco Instruments Inc.; typical radius of curvature ~10 nm and front/side cone angles of 15°/17.5°, respectively).

### 2.3. Three-Electrodes Electrochemical Cell

To allow electrochemical analysis and to complete device packaging, chips were mounted in a custom-built electrochemical cell made of two parts; see Figure 1c. The lower section is composed of an aluminium base supported on a polytetrafluoroethylene (PTFE) base. It contains a central square that is recessed and ca. 0.1 mm larger than the chip dimensions in which it would sit securely during measurements. The upper PTFE cap comprises a sample reservoir and apertures to mount three spring-loaded probes (PM4J micro probe and RM4T-W700 terminal, Coda Systems Ltd. Essex, UK) directly above the contact pads to electrically contact the electrodes to a potentiostat. The sample reservoir can accommodate up to 8 mL of solution and was sealed using a chemically resistant O-ring (Radionics Ltd. Dublin, Ireland.), which is large enough (4.5 mm in diameter) to ensure that all electrodes on the chip have equivalent access to the analyte solution. When fully assembled, the chips were sandwiched between the PTFE sections, which were screwed together, enabling the use of either the on-chip Pt pseudo-reference electrode (see Figure 1d) or an external reference electrode such as standard calomel electrode (SCE) (see Figure 1e).

### 2.4. Nanoporous Gold Modification

NPG modification experiments were performed at gold microdisc array working electrodes vs. an external SCE reference electrode (Dublin Analytical Instruments, Dublin, Ireland) and an on-chip platinum counter electrode. Au_0.18_Ag_0.82_ alloy thin films were electrodeposited onto clean substrates at −1.19 V for 3 s in solutions containing 100 mM KAg(CN)_2_ (99.9%, Alfa Aesar), 20 mM KAu(CN)_2_ (99.99%, Alfa Aesar) and 250 mM Na_2_CO_3_ (99.5%, ACS grade) at pH 13. Following Au_0.18_Ag_0.82_ alloy electrodeposition, NPG structures were obtained by selectively etching the Ag component from the alloy in 25% nitric acid for 2 min. This approach is based on the work of Searson et al. [79,80], who demonstrated that this particular composition of KAu(CN)_2_ and KAg(CN)_2_ resulted in deposition of the particular alloy composition of Au_0.18_Ag_0.82,_ which in turn yielded the largest NPG surface area following etching. Cyclic voltammograms (CVs) in 1 M NaOH from −0.90 to 0.88 V vs. SCE at 10 mV s^−1^ were recorded prior and subsequent to alloy deposition and post-etching to estimate the electrochemically active surface area (and also for quality control purposes).

### 2.5. Electrochemical Analysis

All electrochemical experiments were performed at room temperature (17–20 °C) using a CHI660B potentiostat (CH Instruments, IJ Cambria, Llanelli, United Kingdom.), supplied by IJ Cambria, connected to a personal computer. All experiments employed a standard three-electrode cell configuration using gold- or NPG-modified microdisc arrays as the working electrode and an on-chip platinum counter electrode with an on-chip platinum pseudo-reference electrode or an off-chip SCE reference electrode. CV measurements were performed vs. Pt in 10 mM phosphate buffer saline (PBS, pH 7.4) solution and in 1 mM ferrocene monocarboxylic acid (FCA) in 10 mM PBS solution in an appropriate potential window at 10 mVs^−1^. Cyclic and linear sweep voltammetry studies were performed vs. Pt or vs. SCE in as-received commercial Ventolin^TM^ formulation in an appropriate potential window.

### 2.6. Chemicals and Glassware

Prior to electrochemical experiments, on-chip electrodes were cleaned by sequential immersion for 10 min in acetone and isopropyl alcohol, followed by thorough rinsing with deionised water and drying in a filtered stream of nitrogen, and oxygen plasma-etched for 10 min at 200 W. Unless otherwise stated, all chemicals were purchased from SigmaAldrich and were used as received. Solutions were prepared using deionised water.

## 3. Results and Discussion

### 3.1. Microscopical Characterisation of Gold Microdisc Array Electrode

**Figure 2:**
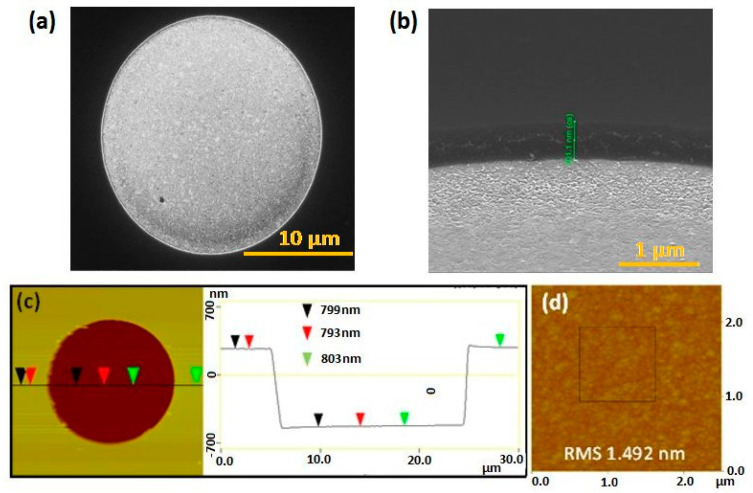
(**a**) Plan view SEM image; (**b**) tilted side view SEM image of an individual gold microdisc; (**c**) AFM micrograph and corresponding topographical section analysis; (**d**) AFM micrograph for roughness analysis.

Following device fabrication, structural characterisation using SEM demonstrated that no residual silicon nitride remained on the microdisc electrodes, i.e., the selective removal of the silicon nitride overlayer to expose the underlying gold surface was complete. Figure 2a shows a characteristic top plan view SEM micrograph of an individual microdisc. Figure 2b is a typical tilted SEM image at higher magnification highlighting the recess depth. Statistical analysis of the SEM data of across 19 wafers representative of 5 separate fabrication runs, several months apart, yielded an average diameter of 23.04 ± 0.34 µm (n = 30) and an average recess depth of 720 ± 109 nm (n = 30), thereby demonstrating the high reproducibility of the fabrication process.

Following the selective removal of the silicon nitride overlayer, topographical analysis was also undertaken by AFM on a few devices (n = 3). An AFM micrograph obtained at a gold microdisc electrode with details of the section analysis is depicted in Figure 2c. The microdisc recess depth was estimated from line scans in the height data to be ~798 nm, which correlates well with the average obtained for multiple microdiscs using SEM analysis. Moreover, AFM studies also yielded topographical information permitting analysis of the roughness of microdisc surface. These studies were conducted in a 1 μm^2^ square area within a 2.5 μm^2^ square scan area, as shown in Figure 2d. The typical RMS roughness of the microdisc surfaces was ca. 1.5 nm. An experimental total surface area of the microdisc was extracted from this topographical analysis. To this end, the roughness factor, ρ, of the microdisc was determined using Equation (1):

ρ = AM/AG(1)

where AM is the measured surface area of the microdisc (~1.5 × 1.5 = 2.25 μm^2^), and AG is the two-dimensional area occupied by a microdisc in a 1 μm^2^ AFM micrograph.

In this manner, the microdisc roughness factor, ρ, was determined to be 2.25. The increased size of the surface area may be attributed to surface roughening of the microdisc electrodes during the etching process to remove the silicon nitride layer. From this analysis, the average electrochemically active area of a typical array of gold microdisc electrode was estimated to be ~5.23 × 10^−^^4^ cm^2^ (geometric area × roughness factor), taking the geometric area as the surface area of 56 discs of 23.04 µm diameter.

### 3.2. NPG Modification of Microdisc Arrays with High Reproducibility

**Figure 3:**
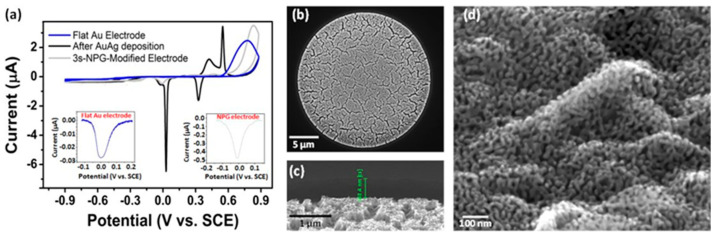
(**a**) CVs in 1 M NaOH at 10 mV s^−1^ recorded at a gold microdisc array (blue), after Au_0.18_Ag_0.82_ electrodeposition (black) and at an NPG-modified one (grey). Insets show gold oxide reduction peaks at bare gold- and NPG microdisc array electrodes (left and right, respectively); (**b**) plan view SEM image; (**c**) tilted SEM image of NPG microdisc; (**d**) high magnification SEM image of NPG structure.

As described in the experimental section, NPG modification experiments were performed at gold microdisc array working electrodes by electrodeposition at −1.19 V vs. SCE for 3 s in a solution of 100 mM KAg(CN)_2_ and 20 mM KAu(CN)_2_ in 250 mM Na_2_CO_3_ at pH 13 to yield Au_0.18_Ag_0.82_, followed by etching of the silver component in 25% nitric acid for 2 min and was based on a process described earlier for modification of arrays of gold microbands and gold microdiscs [66,67]. CVs in 1 M NaOH over the range −0.90 to 0.90 V at 10 mVs^−1^ were recorded vs. SCE in between each step, as shown in Figure 3a.

The CV at the gold microdisc array electrode (blue line) shows that negligible current flows over the potential range from −0.90 to 0.15 V prior to the onset of gold oxide monolayer formation, while, on the reverse scan, the corresponding reduction peak is observed at around −0.03 V. The magnitude of the reduction peak current was found to be highly reproducible across 19 wafers representative of five separate fabrication runs, fabricated several months apart: −30.3 ± 6.4 nA (n = 76). An average electrochemically active area of 1.06 ± 0.71 × 10^−3^ cm^2^ was estimated from the charge associated with the gold oxide reduction peak. This estimation is about twice that obtained previously from AFM characterisation, probably because this technique is based on the assumption of a monolayer oxide formation only with 1:1 stoichiometric conditions, which are difficult to achieve practically at the micron scale; the estimated value may be due to formation of oxide multilayers.

The electrodeposition of Au_0.18_Ag_0.82_ alloys was also found to be highly reproducible, as demonstrated in the chronoamperometric data shown in Figure S1. The shape and the current magnitude of the recorded signal was very consistent from chip to chip and wafer to wafer. Electrodeposition of Au_0.18_Ag_0.82_ involves the reduction of gold and silver ions present in the plating bath at the gold microdisc electrode surface. In its simplest form, it may be described by the following reaction:

Au^+^ (aq) + Ag^+^ (aq) + 2 e^−^→AuAg (s) (2)

The thickness *T* of the electrodeposited layer may be calculated using Equation (3):

(3) $$T=\frac{Q\;M_{wt}}{nFAd}$$

where *Q* is the total charge associated with the electrodeposition, *M_wt_* is the molecular weight of the resulting Au_0.18_Ag_0.82_ alloy (123.91 g.mol^−1^), *n* is the number of electrons involved in the reaction, *F* is Faraday’s constant, *A* is the area of the deposit (given as the electrode geometric area, 2.324 × 10^−4^ cm^2^) and *d* is density of the alloy (12.076 g cm^−3^). An average thickness of 200 ± 42 nm (n = 60) was measured. Based on the average charge associated with the obtained chronoamperograms, the alloy thickness was estimated to be about 188 nm. The CV of the Au_0.18_Ag_0.82_ alloy (Figure 3a) clearly indicated the presence of Au and Ag from the characteristic metal oxide peaks seen at 0.42 V and 0.55 V, respectively, in the anodic region and from the corresponding reduction peaks seen at 0.03 V and 0.32 V, respectively, in the cathodic region. The alloy deposition was highly reproducible, as the current magnitude of the oxidation and reduction peaks were found to be consistent across 19 gold microdisc arrays representative of five separate fabrication runs several months apart (n = 60): 0.92 ± 0.15 µA for Au oxidation, 2.71 ± 0.51 µA for Ag oxidation, −5.08 ± 0.80 µA for Au reduction and −1.33 ± 0.27 µA for Ag reduction. Following immersion of the alloyed microdisc array in 25% nitric acid for 2 minutes, the resulting de-alloyed electrode yields a CV response in 1 M NaOH (shown in grey) that is typical of gold and shows the absence of silver oxidation and reduction peaks, indicating the selective removal of almost all of the silver component. The gold oxide reduction peak current has increased by an order of magnitude, which is associated with a high increase in electrochemical active surface area. This peak current was also found to be highly reproducible across 19 NPG-modified disc arrays representative of five separate fabrication runs several months apart: −296 ± 69 nA (n = 30). An average electrochemically active area of 8.98 ± 0.85 × 10^−3^ cm^2^ was estimated from the charge associated with the gold oxide reduction peak, which is ca. nine times higher than the unmodified gold microdisc array.

SEM characterisation of the modified microdisc array revealed the presence of an NPG layer in thin film format that partially filled the recessed discs. A high magnification image of an individual NPG-modified microdisc shown in Figure 3b highlights that the NPG film has the same diameter as the underlying gold disc. Figure 3c is a high magnification SEM image of a tilted substrate indicating that the NPG film is about 220 nm high, which is in good agreement with the estimation of the film’s height in Figure S1. The sponge-like 3D structure with interconnecting pores of 20–30 nm and ligaments of 30–50 nm that typifies NPG is shown in Figure 3d.

### 3.3. Functionality of Microdisc Array Electrodes

#### 3.3.1. Bare Gold Microdisc Array Electrodes

**Figure 4:**
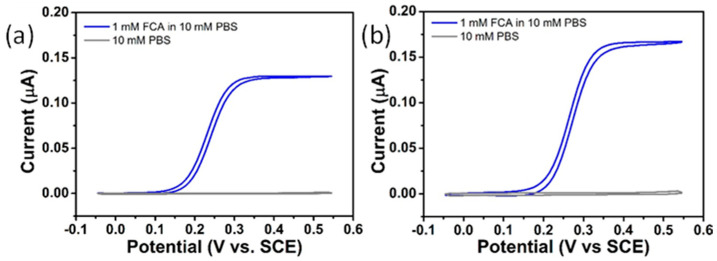
Typical CVs obtained for 1 mM FCA in 10 mM PBS (blue line) and in 10 mM PBS (grey line) at 10 mVs^−1^ at (**a**) gold microdisc array electrode and (**b**) NPG-modified microdisc array electrode.

Following fabrication, CV studies were undertaken by applying an appropriate potential sweep range to the gold microdisc array working electrodes. FCA was selected as the model redox probe molecule for electrochemical assessment of the functionality of the gold microdisc array electrodes. Figure 4a shows typical CVs for 1 mM FCA in 10 mM PBS (shown in blue) and in 10 mM PBS (shown in grey) at 10 mV s^−1^.

In the presence of 1 mM FCA, the measured CVs at 10 mV s^−1^ displayed sigmoidal responses reaching a steady-state current, typical of a single electron oxidation occurring at a micron-scale electrode and consistent with comparable array types previously reported [67]. This confirmed that the performance of the on-chip three-electrode cell was not compromised by the reduced cell volume (~200 μL when using the on-chip pseudo-reference electrode), the on-chip counter electrode dimensions or by electrical contact using spring-loaded probes. The steady-state current was found to be highly reproducible across 19 wafers representative of five separate fabrication runs performed several months apart: 0.141 ± 0.042 µA (n = 24). This further demonstrates the highly reproducible nature of the fabrication process; variations may be associated with the small variations observed in electrodes dimensions. Furthermore, the steady-state current was benchmarked against a theoretical model for recessed microdisc array electrodes that expresses the relationship between the limiting current, *I_lim_*, and the number of microdiscs in Equation (4)

(4) $$\;I_{lim}=N\frac{4\pi nFCDr^{2}}{4L+\pi r}$$

where *n* is the number of electrons involved in the reaction, *F* is Faraday’s constant (96,485 C mol^−1^), C* is the bulk concentration of the FCA (10^−6^ mol cm^−3^), *D*_O_ is the diffusion coefficient (5.4 × 10^–6^ cm^2^ s^–1^), *r* is the disc radius (11.52 × 10^–4^ cm), *L* is the recess depth (720 × 10^–7^ cm) and *N* is the number of microdiscs in an array.

The FCA limiting current was estimated to be 0.157 µA using Equation (2), which is similar to that found experimentally and confirms that individual radial diffusion layers occur at each microdisc in the array. Essentially, the diffusion layers at individual microdiscs are unperturbed by the diffusion layers of the neighbouring electrodes, resulting in enhanced voltammetric performance. Control CV experiments in the absence of FCA were performed in PBS buffer, only, at 10 mV s^−1^ (grey response in Figure 4a). No faradaic current was observed, but a negligible background current (typically 1–2 nA) was measured.

#### 3.3.2. NPG-Modified Gold Microdisc Array Electrodes

Comparable CV studies were undertaken at NPG-modified microdisc arrays; see Figure 4b. The CV in PBS buffer, only, showed that the magnitude of the nonfaradaic current at 10 mV s^−1^ was negligible (ca. 2–3 nA). Similar results were obtained to those at bare gold microdisc arrays; in the presence of 1 mM FCA (blue response), NPG electrodes displayed highly reproducible sigmoidal responses reaching a steady-state current on the order of 0.170 μA. However, despite the significantly enhanced surface area of NPG compared to bare gold, the current magnitude is only ca. 11% higher than that at the bare gold microdisc arrays. The relatively small increase in current, relative to what could be expected for the higher surface area NPG, can be attributed to the fast electron transfer kinetics of the diffusion-controlled reaction of the ferri-/ferrocyanide redox probe. This results in the reaction occurring primarily on the outermost surface of NPG due to only partial penetration of the redox probe into the nanoporous channels. Thus, the peak current is dependent on the diffusion coefficient of the redox probe: the overlapping diffusion zones between adjacent nanopores creates a limiting semi-infinite linear diffusion field, and the current density is established predominantly by the geometric surface area [81]. Estimation of the electrochemically addressable electrode surface area of NPG is better achieved using the charge required to strip a gold oxide layer, as this is a kinetically-controlled, surface-confined redox reaction, rather than using the FCA redox probe.

### 3.4. Detection of Salbutamol API and Chloride Excipient in Ventolin^TM^ at Bare Gold and NPG Electrodes

#### 3.4.1. CV of Ventolin^TM^ at NPG Electrodes

**Figure 5:**
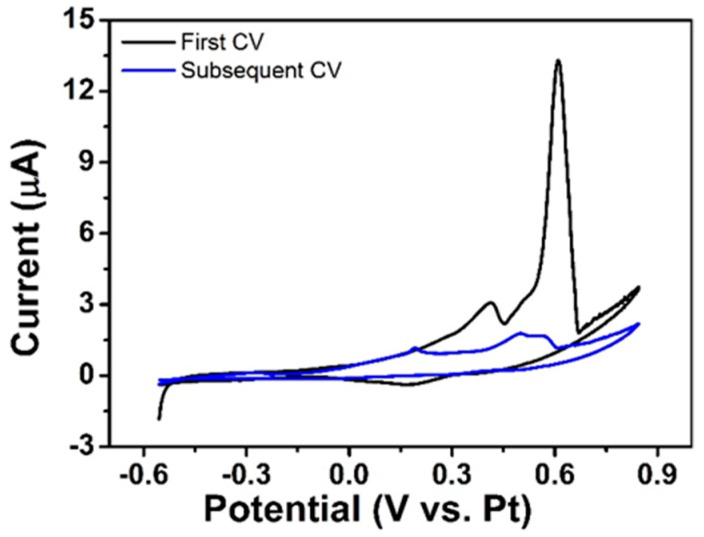
First and second CVs of Ventolin^TM^ formulation at NPG-modified microdisc array electrode recorded from −0.55 to 0.85 V vs. Pt at 10 mV s^−1^.

The CV response of an as-received Ventolin^TM^ formulation containing 6.94 mM (2 mg/mL) salbutamol (as sulphate) in 15.4 mM NaCl and 0.05 mM H_2_SO_4_ solution at pH 4 recorded at an NPG-modified microdisc electrode using the on-chip Pt reference electrode is shown in Figure 5. Two well-defined peaks were identified in the initial anodic scan at 0.41 and 0.61 V, but no corresponding cathodic peaks were observed on the reverse scans, which would suggest that an irreversible reaction, or an electrochemical reaction followed by a chemical step (EC), was associated with each peak. The minor peak at ca. 0.15 V was most likely related to the reduction of gold oxide formed at potentials above 0.70 V. The peaks at 0.41 V and 0.61 V are assigned to chloride and salbutamol oxidation, respectively, with the peak separation of 0.20 V being sufficiently large enough to distinguish the presence of these two species. Second and subsequent CVs demonstrated significant decay in current (blue response). This is in agreement with studies of salbutamol oxidation reported in the literature, which describe an irreversible one electron, one proton oxidation reaction coupled to subsequent fouling of the electrode surface by salbutamol’s oxidation product [34].

**Figure 6:**
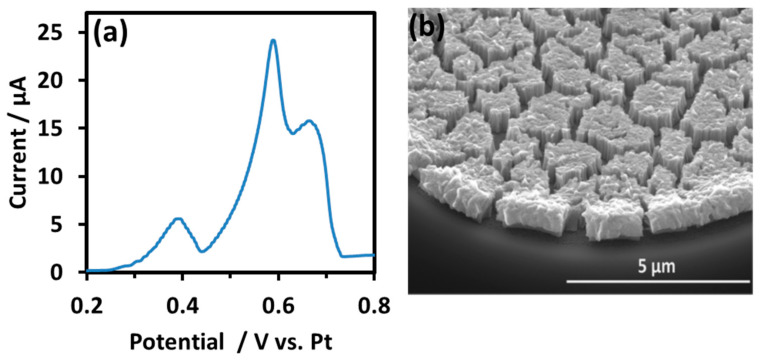
(**a**) LSV of 4 mg/mL salbutamol (as sulphate) in 0.0154 M NaCl and 0.05 mM H_2_SO_4_ solution at pH 4 recorded from 0.20 to 0.80 V vs. Pt at 10 mV s^−1^ at NPG-modified microdisc array; (**b**) tilted view of NPG-modified microdisc following 10 s deposition of Au_0.18_Ag_0.82_ and 6 min etching in 25% nitric acid.

However, it was observed in the linear sweep voltammogram (LSV) for a higher salbutamol concentration of 4 mg/ml performed at a thicker NPG modification layer that the peak associated with salbutamol oxidation could be partially resolved into two peaks at 0.59 V and 0.67 V, as is evident in Figure 6a. The SEM image of a typical NPG-modified disc in the associated electrode array is shown in Figure 6b, wherein the NPG deposit height in the centre of the microdisc is 795 nm (vs. 200 nm in Figure 5). This suggests that the resolution of two stages in salbutamol’s oxidation at NPG is possible under optimized conditions. A more in-depth investigation of salbutamol oxidation in the future using square wave voltammetry (SWV) could yield greater resolution but was not performed herein.

The mechanism of salbutamol electrooxidation, and its associated electron transfer kinetics, has been the subject of intense investigation at a range of electrodes listed earlier in Table 1 [1,34,35,36,37,38,39,40,41,42,43,44]. Most groups reported the appearance of a single peak in the oxidation of salbutamol, which was shown to be indicative of an adsorption-controlled process at some electrodes [1,35,36,41] but as a diffusion-controlled process at a smaller number of electrodes [34,39,44], as substantiated by the linear plots of peak current versus scan rate for the adsorption-controlled processes and peak current versus the square root of scan rate for the diffusion-controlled processes.

Interestingly, Goyal et al. [34] also identified two peaks in the oxidation of salbutamol at indium tin oxide-modified with 4 nm gold nanoparticles in both SWV and differential pulse voltammetry analysis (although LSV failed to detect oxidation peaks at this electrode). The first peak at lower potential was attributed to oxidation of salbutamol in a diffusion-controlled electrode reaction, and the plot of the peak current function (i_p_/ʋ^1/2^) versus ʋ^1/2^ was constant. The second peak, at higher potential, exhibited different behaviour and was attributed to the adsorption of the product formed in the first peak; initially, its peak current function increased with increasing ʋ^1/2^ but then decreased, and such behaviour is suggestive of strong adsorption of the oxidation product [82]. These assertions were substantiated in their GC–MS data, which confirmed that the first peak originates from oxidation of the phenolic hydroxyl group in salbutamol and the second to the strong adsorption of the product formed in this reaction. The oxidation of the phenolic hydroxyl group via a single electron, single proton reaction is well reported in the literature for several compounds, including phenol and 4,4′-biphenol [83,84]. The formation of a C–C linked dimer observed in the GC–MS data can be attributed to the oxidation of the phenolic hydroxyl group to a free radical species, which can subsequently dimerise and adsorb on the electrode. The formation of a similar dimer has also been suggested by Karuwan et al. [85] during oxidation of salbutamol at boron-doped diamond electrode. In a square wave adsorptive stripping voltammetric study, they determined a single oxidation peak over the pH range 3–6, which could be resolved into two peaks at pH between 6–10. The less anodic peak was ascribed to the oxidation of salbutamol, and the more anodic one to the adsorption of its oxidation product.

#### 3.4.2. LSV of Excipients in Ventolin^TM^ at Bare Gold and NPG Electrodes

**Figure 7:**
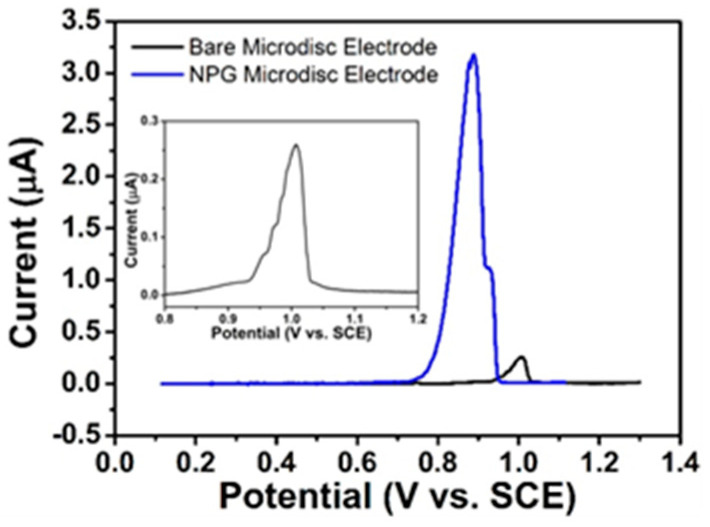
LSVs at bare gold (black)- and NPG-modified microdisc array (blue) electrode arrays vs. SCE at 10 mV s^−1^ in 0.0154 M NaCl and 0.05 mM H_2_SO_4_ background solution (inset shows the response at bare gold microdisc array).

LSV studies in the background solution (i.e., 0.0154 M NaCl and 0.05 mM H_2_SO_4_) at bare gold- and NPG-modified microdisc array electrodes were performed vs. SCE over a suitable potential range at 10 mV s^−1^. As can be seen in Figure 7, a single peak tentatively assigned to the adsorption of chloride ions was recorded in the background solution at 1.005 V and 0.88 V at bare gold and at NPG electrodes, respectively.

It has been well established that chloride ions form a polar chemisorption bond with gold [86,87,88]. Baker et al. reported theoretical evidence from first-principles density functional theory (DFT) calculations that the bonding between a Au(111) surface and chlorine is primarily covalent in character [87]. Although generally the interaction of chlorine with metals is ionic in nature, the Au/Cl system appears to form an exception. Chlorine adsorption on gold is unusual, because gold has the highest Pauling electronegativity of all the transition metals, increasing the likelihood of covalent bond formation with highly electronegative chlorine. The electronegativity difference between Au and Cl of 0.76 is small enough to result in the formation of covalent bonds.

Moreover, the nature of the chloride interaction at gold depends on the nature of the metal’s surface structure. The possible binding sites for chlorine on a gold surface include both flat terrace sites and defect sites. It was shown in a DFT study of the chlorine interaction with Au(111) [88] that chlorine preferentially binds on vacancies, steps and gold adatoms. The binding energy per chlorine atom increased relative to that on a defect-free, flat surface by 0.19 eV when adjacent to a surface vacancy, 0.29 eV at a gold adatom and 0.38 eV at the edge of a step. The release of gold upon chloride adsorption originates from stabilization of adatoms and vacancies.

The 13-fold increase in current for chloride adsorption at NPG (3.4 nA) over bare gold (0.26 nA) noted in Figure 7 cannot solely be attributed to the increase in the electrochemical surface area of NPG (this was estimated to be ca. nine times that of bare gold). The enhanced electrocatalytic activity at low-coordinated sites, grain boundaries, stepped sites and kinks [57,59], whose presence maintain the high radial curvature of the 3D structure of NPG, play a significant role, in combination with the increased binding energy of chlorine at such sites. The cathodic shift in the chloride adsorption peak by 0.125 V upon moving from bare gold to NPG electrodes may be attributed to an embellishment of the electron transfer kinetics for the kinetically controlled adsorption of chloride at this 3D nanoporous architecture. This reveals the dependence of the reaction on the nature of the electrode surface and the role of its atomic structure in modulating reaction efficacy.

The promotion of electron transfer kinetics has been reported in the literature for several other molecules at NPG that undergo kinetically sluggish electron transfer reactions, as evidenced by the cathodic shift in potential for oxidation reactions (and anodic shift for reduction reactions) at NPG relative to bare gold [75,76,89,90,91,92,93]. Most recently, Kumar et al. [89] noted the enhancement in electron transfer kinetics of ascorbic acid at NPG over bare gold (the oxidation wave shifted by 0.30 V to less positive potential at NPG relative to bare gold), which they attributed to the porosity of NPG and to the evolution of low index and higher surface energy crystalline planes. Moreover, the presence of (220), (200) and (311) planes were confirmed at higher intensity at NPG by XRD, which is expected for materials with a higher density of electrocatalytically active sites. The evolution of such low indexed crystalline planes in NPG is in contrast to the (111) plane exposed in bare gold. Similarly, the onset potential for oxidation of glucose, galactose and mannose were approximately 0.10 V lower at Au(100)-enhanced NPG than at bare gold in a recent study [90]. The authors further highlighted the influence of crystallographic orientation on the onset potential for the monosaccharide oxidation reactions, with a lower onset potential at Au(100)-enhanced NPG than at Au(111)–NPG. Much earlier, the enhanced electron transfer kinetics for oxidation of surface-confined nitrite and L-cysteine at NPG relative to bare gold were evidenced in the cathodic shift in their oxidation potentials by Ge et al. [70] and Liu et al. [91], respectively. Similarly, in the case of the reduction of hydrogen peroxide and oxygen, an anodic shift in potential was recorded at NPG relative to bare gold [92,93].

The origin of the potential shift for chloride at NPG observed herein, and for the aforementioned molecules, may be rationalised on the basis of the *nanoconfinement effect*, which was pioneered by Park et al. [94]. This phenomenon bears particular relevance in the case of surface-confined reactants with sluggish electron transfer kinetics that are less influenced by mass transport than those undergoing fast electron transfer with mass transport limitations. Essentially, nanoporous electrodes are predisposed to or biased towards redox couples that have slow rates of electron exchange. Specifically, Park et al. [94] noted that the electrical field gradient and molecular dynamics at nanoporous solid–fluid interfaces are theoretically predicted to be different from those on flat ones. In fact, nanoporous electrodes can even augment the faradaic current for very sluggish electrochemical reactions up to a level expected from diffusion-controlled systems. Essentially, for a reactant undergoing slow electron transfer, which is present at high concentration, the faradaic current generated is comparable to that of one at the same concentration undergoing fast electron transfer at nanoporous electrodes. In the case of chloride that undergoes a sluggish electron transfer reaction, it is likely to prevail at a sufficiently high concentration in the deeper parts of the pores instead of being just confined to the outermost region of NPG. In this situation, electron transfer occurs on almost the entire available external surface and internal pores of NPG. By virtue of chloride residing within a distance shorter than the pore radius from the inner wall surface of NPG, the charge transfer probability between it and the surface is statistically higher. Consequently, heterogeneous charge transfer is facilitated, and the electrode potential reaches equilibrium faster. This is realised as a selective amplification of the amperometric response for the sluggish reaction of chloride at NPG.

The adsorption of chloride at gold is, in fact, of general interest even outside of this study and can present a significant issue in the electrochemical oxidation of glucose at gold electrodes. Nanostructured gold electrode surfaces have been widely utilized in developing electrochemical sensors for glucose due to their high electrochemical activity towards glucose oxidation, but overcoming deactivation of electrode activity in the presence of chloride ions in physiological media (e.g., ca. 0.10 M in blood) is an issue, owing to the inhibition of glucose electrooxidation by adsorbed chloride ions [95]. The oxidation current for a series of monosaccharides glucose and mannose (and, to a lesser extent, galactose) at NPG were shown to decrease significantly in the presence of chloride by Mie et al. [90]. However, in comparison to flat Au surfaces, some nanostructured gold surfaces have been shown to exhibit higher electrochemical activity towards glucose oxidation in the presence of chloride ions [96,97,98,99,100], particularly nanoporous gold, which exhibited a wider linear range and better sensitivity for glucose detection under physiological conditions containing chloride ions [97].

#### 3.4.3. LSV of Ventolin^TM^ at Bare Gold and NPG Electrodes

**Figure 8:**
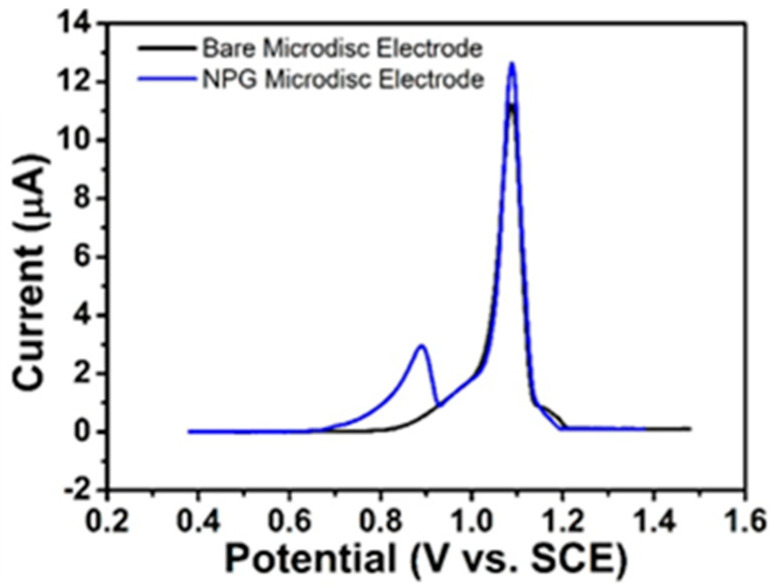
LSVs at bare gold (black)- and NPG-modified microdisc array (blue) electrode arrays vs. SCE at 10 mV s^−1^ in Ventolin^TM^ formulation (pH 4) containing 2 mg/mL salbutamol (as sulphate) in 0.0154 M NaCl and 0.05 mM H_2_SO_4_.

LSV studies in Ventolin^TM^ formulation (pH 4) containing 6.94 mM (2 mg/mL) salbutamol (as sulphate) in 0.0154 M NaCl and 0.05 mM H_2_SO_4_ solutions at bare gold- and NPG-modified microdisc array electrodes were performed vs. SCE over a suitable potential range at 10 mV s^−1^. In Ventolin^TM^ solution, only one anodic peak was observed at bare gold microdisc array electrodes at 1.09 V, whereas two peaks were recorded at NPG-modified array electrodes at 0.88 V and 1.09 V; see Figure 8. This highlights that salbutamol is indistinguishable from chloride at bare gold electrodes, as the potential for salbutamol oxidation and chloride adsorption are very similar. In contrast, the two well-resolved peaks observed at NPG show that salbutamol can be successfully discriminated from chloride (the peaks at 0.88 V and 1.09 V are associated with chloride adsorption and salbutamol oxidation, respectively). However, the salbutamol peak current at NPG (12.8 µA) is only 14% higher than that at bare gold electrodes (11.2 µA), despite the enhanced surface area of NPG. This can be explained in terms of faster electron transfer kinetics of salbutamol oxidation inhibiting its ingress to the internal pores of NPG so that the entire electrode does not participate in the reaction but is confined to its outer surface.

As highlighted earlier, salbutamol oxidation was reported to undergo a diffusion-controlled reaction at gold nanoparticle-modified ITO [34] and other electrodes [39,44]. The fast electrochemical oxidation occurs mostly in the outermost region of the pores, as the electron transfer is much faster in this region than the mass transport of salbutamol molecules from the bulk solution. The faster electrochemical reaction kinetics of salbutamol relative to chloride results in its greater depletion, and, hence, the nanoconfinement effect is far less significant for salbutamol.

#### 3.4.4. Selective Amplification Verified at Thicker NPG Electrodes

**Figure 9:**
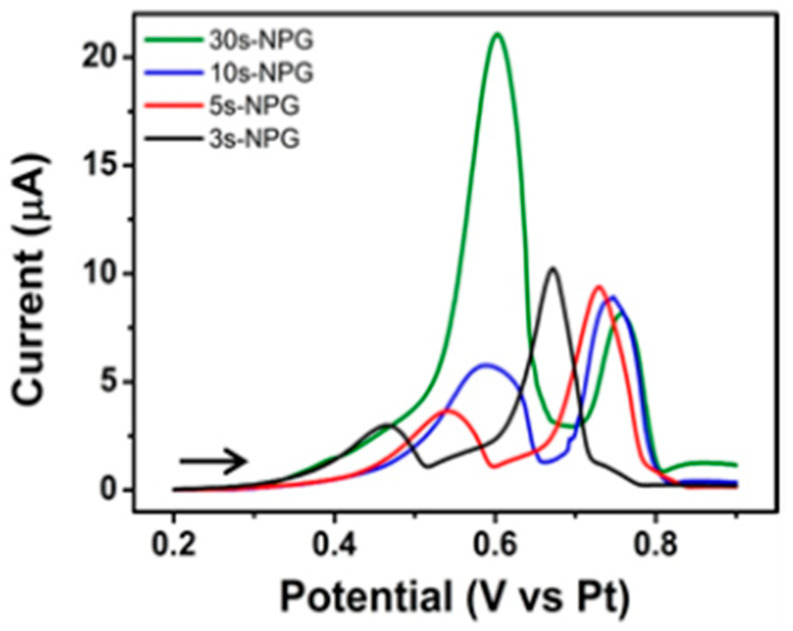
LSV at NPG-modified gold microdisc array electrodes at 10 mV s^−1^ in Ventolin^TM^ solutions (pH 4) containing 2 mg/mL salbutamol (as sulphate) in 0.0154 M NaCl and 0.05 mM H_2_SO_4_ following 3, 5, 10 and 30 s deposition of Au_0.18_Ag_0.82_ alloy and subsequent etching in nitric acid to yield NPG.

**Figure 10:**
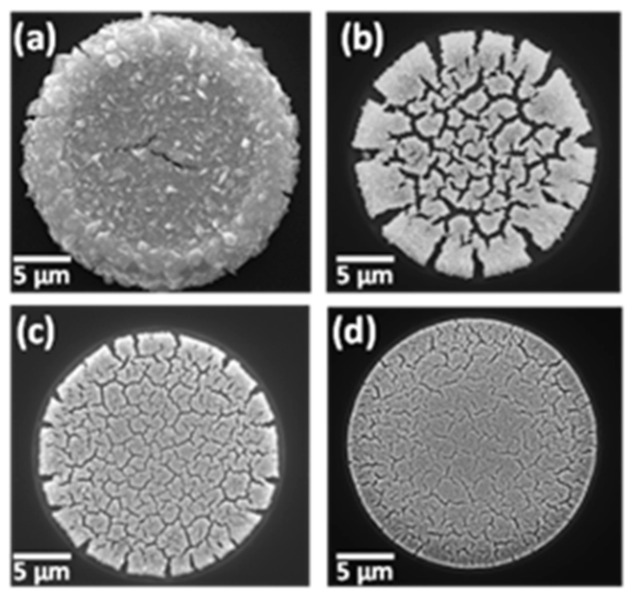
Plan view SEM images of a NPG-modified microdisc following (**a**–**d**) 30, 10, 5 and 3 s deposition of Au_0.18_Ag_0.82_ alloy NPG and subsequent etching in nitric acid to yield NPG.

The differing response of NPG to salbutamol and chloride as a function of their intrinsic electron transfer rates is another example of the ‘discriminative’- or ‘selective current amplification’ previously attributed to nanoporous electrodes [101]. The nanoconfinement effect associated with chloride leading to its selective amplification at NPG was confirmed experimentally in Figure 9 by varying the NPG deposit thickness (and, hence, its available surface area) in the microdisc array and observing the change in the LSV response for salbutamol and chloride in Ventolin^TM^ formulations. The current associated with chloride adsorption increased with NPG deposit thickness, yet the salbutamol oxidation current remained almost constant. It should be noted that there is some variability in the peak potential for salbutamol and chloride signals due to the slight instability of the platinum quasi-reference electrode. The SEM images corresponding to the NPG-modified microdisc arrays of varying thickness are shown in Figure 10. Deposition of the precursor Au_0.18_Ag_0.82_ alloy for 30, 10, 5 and 3s, followed by etching in 25% nitric acid for 30, 6, 3 and 2 min, respectively, yielded NPG deposits of 1.6, 1.0, 0.7 and 0.28 µm thickness, respectively. The deposition was found to be affected by the shape of the microdisc such that somewhat thicker deposits formed at the periphery of the microdisc while thinner deposits formed in the central area. This is due to differences in the current flow; it flows more densely to sharp edges than to the less accessible recessed areas.

The selective amplification of the chloride reaction over that of salbutamol at NPG can be harnessed to confer high selectivity to the discrimination of the salbutamol signal from that of chloride interference, which is not feasible at planar gold. Guntapalli et al. [102] also observed the simultaneous detection and discrimination of dopamine and serotonin in a mixture at porous gold–single-walled carbon nanotube hybrid film with a resolution greatly exceeding that at planar gold. Similar to the behaviour observed for salbutamol and chloride herein, they attributed the enhanced resolution to the response of porous gold to differing rates of electron transfer of multiple species stemming from negligible mass transfer and shorter diffusion time for molecules in the pores. An amplification of serotonin oxidation relative to dopamine was reported due to the slower electron transfer of the former. This serves to further highlight the potential of harnessing nanogeometrical control in NPG to achieve differentiation and/or electrocatalytic enhancement of analytes in complex mixtures and in biological samples.

#### 3.4.5. Nature of Salbutamol and Chloride Reactions at NPG Electrodes

It was confirmed in Figure S2 that the current for the single peak associated with salbutamol oxidation and chloride adsorption at bare gold electrodes increased linearly with scan rate over the range 20–100 mVs^−1^, which is indicative of adsorption-control, but deviations from linearity were found for scan rates below 20 mV s^−1^. A linear plot of the log of peak current versus log scan rate was obtained with slope of 0.23, as shown in Figure S3. A slope of less than 0.5 is associated with a mixed adsorption-diffusion process, as would be expected for the oxidation of salbutamol (diffusion-controlled oxidation followed by product adsorption) and an adsorption-controlled reaction for chloride [34].

At NPG-modified microdisc array electrodes, the analysis of peak current with scan rate for the chloride peak confirmed an adsorption-controlled process, based on the linear plot of peak current versus scan rate shown in Figure S4. Analysis of the peak current for salbutamol was more complex; the peak current function (i_p_/ʋ^1/2^) increased initially with the square root of scan rate and then decreased (Figure S5). This behaviour is suggestive of strong adsorption of the product formed in the oxidation of salbutamol at NPG [84]. Moreover, analysis of the variation of salbutamol peak potential with scan rate shown in Figure S6 showed that the peak potential (E_p_) increased with increasing scan rate, thereby confirming the irreversibility of the reaction. The slope of the straight line plot of E_p_ versus log of scan rate was found to be 0.162 V/decade, which is far greater than the expected value of 0.059 V/decade for a simple 1 electron irreversible reaction and provides further affirmation of the assertion that the reaction follows an EC mechanism [103], which involves an electron transfer step (E) followed by a chemical reaction (C). Essentially, salbutamol undergoes an irreversible electrochemical oxidation reaction, and its dimeric product undergoes a chemically irreversible reaction at NPG. SEM analysis revealed the presence of an adsorbed layer on NPG after recording an LSV in Ventolin^TM^, as shown in Figure S7.

#### 3.4.6. Quantification of Salbutamol at NPG Electrodes

**Figure 11:**
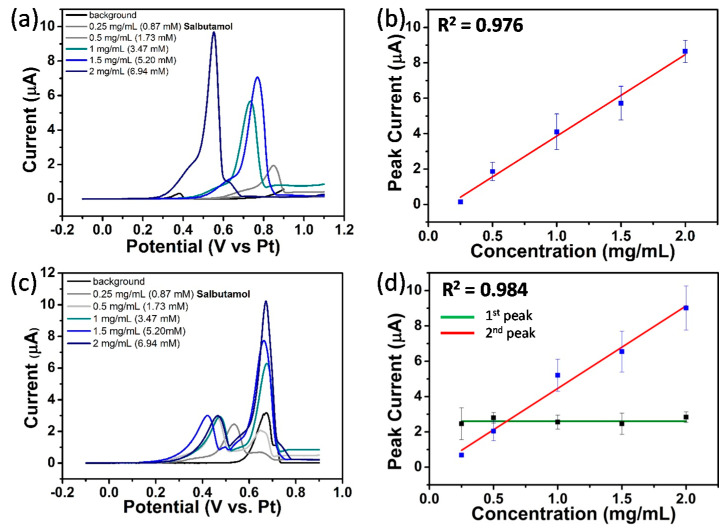
(**a**) LSVs at bare gold microdisc array electrodes at 10 mV s^−1^ in background solution (pH 4) and in Ventolin^TM^ solutions (pH 4) containing 0.25, 0.5, 1, 1.5 and 2 mg/mL salbutamol (as sulphate) in 0.0154 M NaCl and 0.05 mM H_2_SO_4_; (**b**) plot of peak current vs. salbutamol concentration at bare gold electrodes; (**c**,**d**) are the equivalent of (**a**,**b**) at NPG electrodes.

LSV studies in Ventolin^TM^ formulation (pH 4) containing 0.87 to 6.94 mM (0.25 to 2 mg/mL) salbutamol (as sulphate) in 0.0154 M NaCl and 0.05 mM H_2_SO_4_ solutions at gold electrodes were performed vs. on-chip Pt reference electrode over a suitable potential range at 10 mVs^−1^; see Figure 11a. Only one oxidation peak was observed. The slight instability of the platinum quasi-reference electrode accounts for the variability in the peak potential for salbutamol and chloride signals for different concentrations but serves to demonstrate the advantage of using an integrated reference electrode over that of an external one. It was found that the peak current increased linearly with increasing salbutamol concentration, with an average linear regression equation (R^2^ = 0.976) of I_pa_ (µA) = 1.53 C_salbutamol_ (mM) − 0.68 and a detection limit of 0.26 mM; see Figure 11b. The same set of experiments were conducted at NPG microdisc electrode arrays; see Figure 11c. Two oxidation peaks were recorded for each solution. The first peak at ca. 0.40 V, previously attributed to chloride_,_ was found to vary only slightly with varying solution, while the current for the second peak at ca. 0.70 V increased linearly with increasing salbutamol concentration. An average linear regression (R^2^ = 0.984) of I_pa_ (µA) = 1.35 C_salbutamol_ (mM) − 0.02 and a detection limit of 0.17 mM was found; see Figure 11d. Consequently, NPG electrodes display enhanced performance compared to their bare gold counterpart in terms of discrimination between the different ingredients and lower salbutamol detection limit. Analysis was performed in triplicate using three different substrates, and the calibration curves were constructed using the average peak curve of such measurements. The concentration range investigated was confined to 0.25 mg/mL to 2 mg/mL salbutamol in this study; hence, the magnitude of the linear range that is attainable and the lowest possible detection limit remains undetermined for salbutamol at this sensor.

## 4. Conclusions

The present study provides insights into the potential of harnessing NPG’s unique nanogeometrical properties, enhanced electrocatalytic activity and increased density of low-indexed crystalline planes and high specific surface area NPG to selectively discriminate an API from its excipients in pharmaceutical formulations. The signals from the API in Ventolin^TM^ formulations and its chloride excipient were resolved at NPG-modified microdisc arrays by exploiting the difference in the kinetics of their electron transfer reactions and the increased density of active sites in NPG. Superior selectivity and lower detection limits than that attained at bare gold microdisc arrays were realized.

In future work at NPG electrodes, SWV could be applied to the determination of salbutamol in Ventolin^TM^ formulations. Exploiting its potential to effectively discriminate the faradaic current from the charging could permit better resolution of the two peaks associated with salbutamol oxidation at NPG, increase the detection sensitivity and reduce the lower detection limit.

Furthermore, the role of particular crystallographic orientations in NPG (especially for low-index planes) in determining the efficiency of electrocatalysis of the reactions associated with a mixture of API(s) and excipients in pharmaceutical formulations and in other complex matrices warrants further investigation.

## Figures and Tables

**Table 1:** This table gives an overview of electrochemical sensors applied to the voltammetric detection of salbutamol.

Electrode	Linear Range/µM	Detection Limit	References
Single-walled carbon nanotube-modified pyrolytic graphite	0.15–7.5	0.129 µM	[34]
Gold nanoparticle–polythionine-modified electrode	0.2–200	0.6 pM	[35]
Graphene–Au nanocomposite	0.05–200	Not specified	[36]
Iron titanate-modified carbon paste electrode	0.0002–0.025	90 pM	[37]
Ag/N-doped reduced graphene oxide incorporated with molecularly imprinted polymer	0.03–20	7 nM	[38]
Graphene/PEDOT:PSS-modified screen printed carbon electrode	0.01–1.20	100 pM	[39]
Cathodically pretreated boron-doped diamond	17.3–347	5 µM	[40]
Poly(4-amino-3-hydroxynaphthalene sulfonic acid)-modified glassy carbon electrode	0.2–8.0	0.6 pM	[41]
MnO_2_ nanoflowers-modified 3D RGO/Ni foam	0.042–1.463	23 nM	[42]
Gold nanoparticle-modified indium tin oxide electrode	0.15–6.0	0.225 µM	[43]
Graphene–ionic liquid silver nanoparticle composite	0.079–2.9	13 nM	[44]
Multiwalled carbon nanotubes and poly(pivalic acid)-modified glassy carbon electrode	0.05–70	12 nM	[45]

## Supplementary Materials

The following are available online at https://www.mdpi.com/article/10.3390/s21123975/s1. Figure S1: Chronoamperograms recorded at five different gold microdisc array electrodes during 3 s AuAg electrodeposition at −1.19 V vs. SCE in a solution of 100 mM KAg(CN)_2_ and 20 mM KAu(CN)_2_ in 250 mM Na_2_CO_3_ at pH 13 at 20 °C. Figure S2: Plot of peak current versus scan rate for the peak associated with combined salbutamol oxidation and chloride adsorption in 2 mg/mL salbutamol in 0.0154 M NaCl and 0.05 mM H_2_SO_4_ solution at pH 4. Figure S3: Plot of log of peak current versus log scan rate for the peak associated with combined salbutamol oxidation and chloride adsorption in 2 mg/mL salbutamol in 0.0154 M NaCl and 0.05 mM H_2_SO_4_ solution at pH 4. Figure S4: Plot of peak current versus scan rate for chloride oxidation in 2 mg/mL salbutamol in 0.0154 M NaCl and 0.05 mM H_2_SO_4_ solution at pH 4 taken from the LSV recorded from −0.20 to 1.0 V vs. Pt at 10 mV s^−1^ at NPG-modified gold microdisc array electrode. Figure S5: Plot of peak current function for salbutamol versus square root of scan rate recorded in 2 mg/mL salbutamol in 0.0154 M NaCl and 0.05 mM H_2_SO_4_ solution at pH 4 taken from the LSV recorded from −0.20 to 1.0 V vs. Pt at 10 mV s^−1^ at NPG-modified gold microdisc array electrode. Figure S6: Plot of peak potential for salbutamol oxidation versus log of scan rate recorded in 2 mg/mL salbutamol in 0.0154 M NaCl and 0.05 mM H_2_SO_4_ solution at pH 4 taken from the LSV recorded from −0.20 to 1.0 V vs. Pt at 10 mV s^−1^ at NPG-modified gold microdisc array electrode. Figure S7: Plan view SEM image of NPG-modified microdisc array following recording of LSV from −0.20 to 1.0 V vs. Pt at 10 mV s^−1^ in 1 mg/mL salbutamol in 0.0154 M chloride and 0.05 mM H_2_SO_4_.
