# Supplementary material for: Electrochemical Discrimination of Salbutamol from Its Excipients in VentolinTM at Nanoporous Gold Microdisc Arrays
Source: Sensors (Basel). 2021 Jun 9;21(12):3975. doi: 10.3390/s21123975 (PMC8226559; doi:10.3390/s21123975)
Supplement: Supplementary file 1 [file sensors-21-03975-s001.zip › sensors-1224855-SI.pdf]

Supplementary Materials

Electrochemical Discrimination of Salbutamol from its Excipients in Ventolin™ at Nanoporous Gold Microdisc Arrays

Lorraine C. Nagle [1]*, Amelie Wahl[1], Vladimir Ogourstov[1], Ian Seymour[1], Fiona Barry[1], James F. Rohan[1] and Ronan Mac Loughlin [2]

[1]**Tyndall National Institute, Lee Maltings Complex, University College Cork, Ireland, T12R5CP**

[2]**Science and Emerging Technologies, Aerogen Ltd. Galway Business Park, Dangan, Galway, Ireland**

* **Correspondence lorraine.nagle@tyndall.ie; Tel.: (+353-21-4904266)**

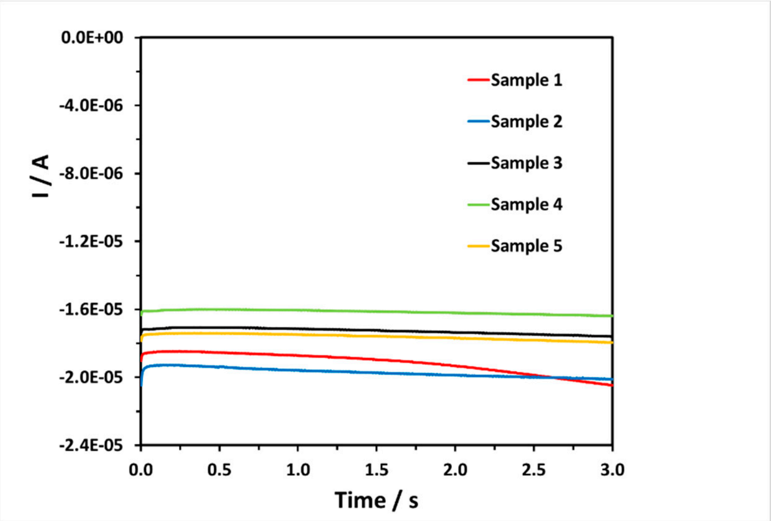

Figure S1. Chronoamperograms recorded at five different gold microdisc array electrodes during 3 s AuAg electrodeposition at -1.19 V vs. SCE in a solution of 100 mM $KAg(CN)_2$ and 20 mM $KAu(CN)_2$ in 250 mM Na_2CO_3 at pH 13 at 20 ^oC.

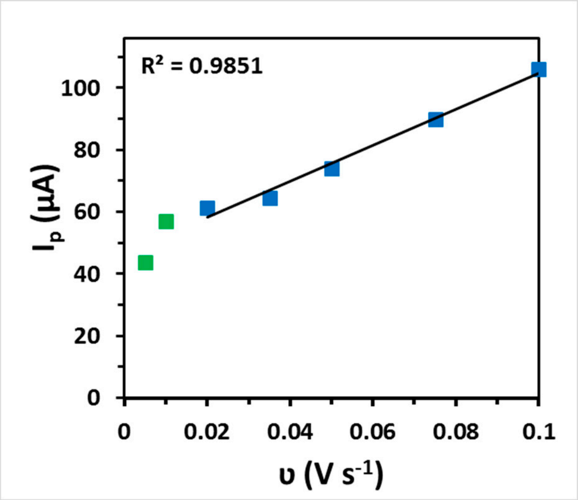

Figure S2. Plot of peak current versus scan rate for the peak associated with combined slbutamol oxidation and chloride adsorption in 2 mg/ml salbutamol in 0.0154 M NaCl and 0.05 mM H_2SO_4 solution at pH 4. The peak current was taken from the LSV recorded from -0.2 to 1.0 V vs. Pt at 10 mV s^{-1} at bare gold microdisc array electrode.

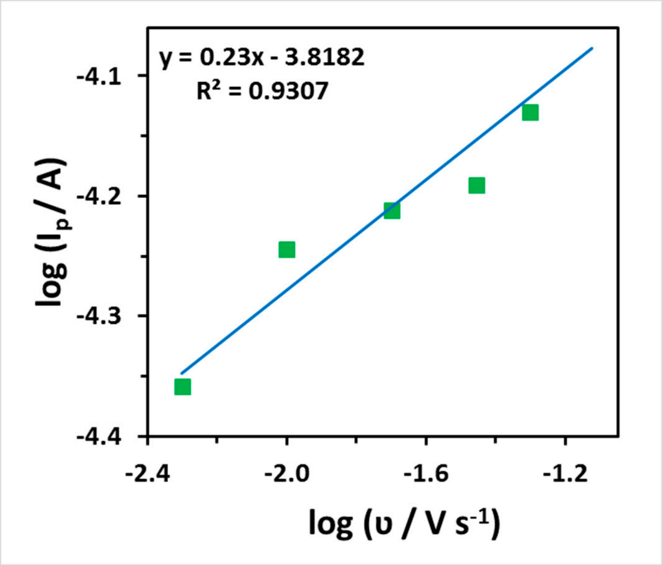

Figure S3. Plot of log of peak current versus log scan rate for the peak associated with combined salbutamol oxidation and chloride adsorption in 2 mg/ml salbutamol in 0.0154 M NaCl and 0.05 mM H_2SO_4 solution at pH 4. The peak current was taken from the LSV recorded from -0.20 to 1.0 V vs. Pt at 10 mV s^{-1} at a bare gold microdisc array electrode.

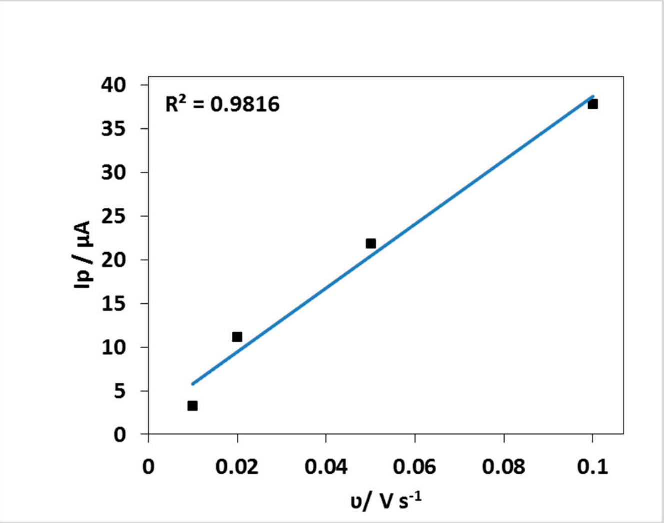

Figure S4. Plot of peak current versus scan rate for chloride adsorption in 2 mg/ml salbutamol in 0.0154 M NaCl and 0.05 mM H_2SO_4 solution at pH 4 taken from the LSV recorded from -0.20 to 1.0 V vs. Pt at 10 mV s^{-1} at NPG-modified gold microdisc array electrode.

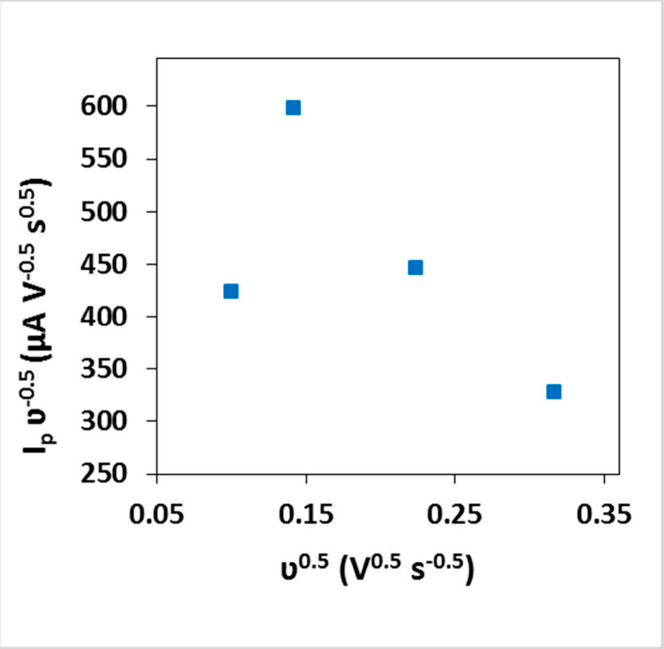

Figure S5. Plot of peak current function for salbutamol oxidation versus square root of scan rate recorded in 2 mg/ml salbutamol in 0.0154 M NaCl and 0.05 mM H_2SO_4 solution at pH 4 take from the LSV recorded from -0.20 to 1.0 V vs. Pt at 10 mV s^{-1} at NPG-modified gold microdisc array electrode.

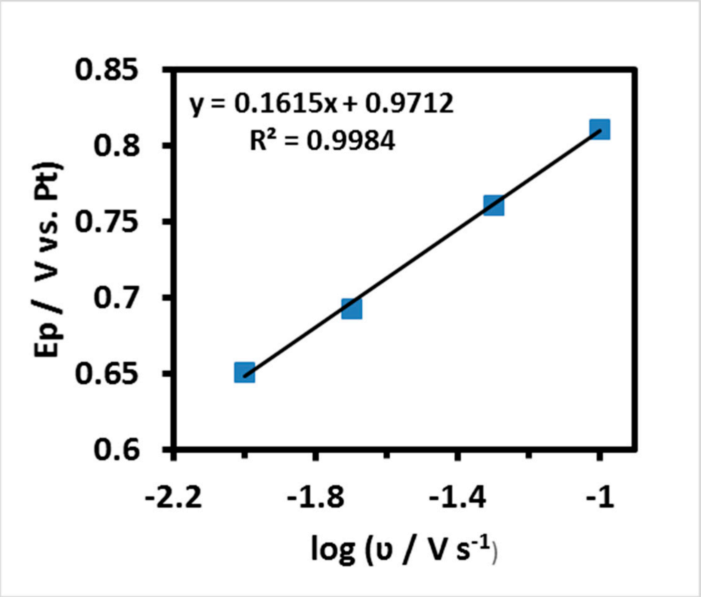

Figure S6. Plot of peak potential for salbutamol oxidation versus log of scan rate recorded in 2 mg/ml salbutamol in 0.0154 M NaCl and 0.05 mM H_2SO_4 solution at pH 4 taken from the LSV recorded from -0.20 to 1.0 V vs. Pt at 10 mV s^{-1} at NPG-modified gold microdisc array electrode.

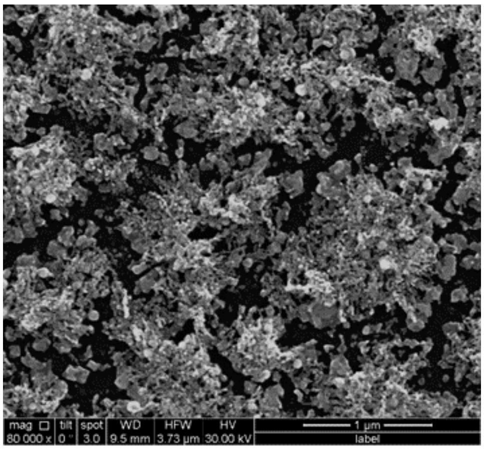

Figure S7. Plan view SEM image of NPG-modified microdisc array following recording of LSV from -0.20 to 1.0 V vs. Pt at 10 mV s^{-1} in 1 mg/ml salbutamol in 0.0154 M chloride and 0.05 mM H_2SO_4.
